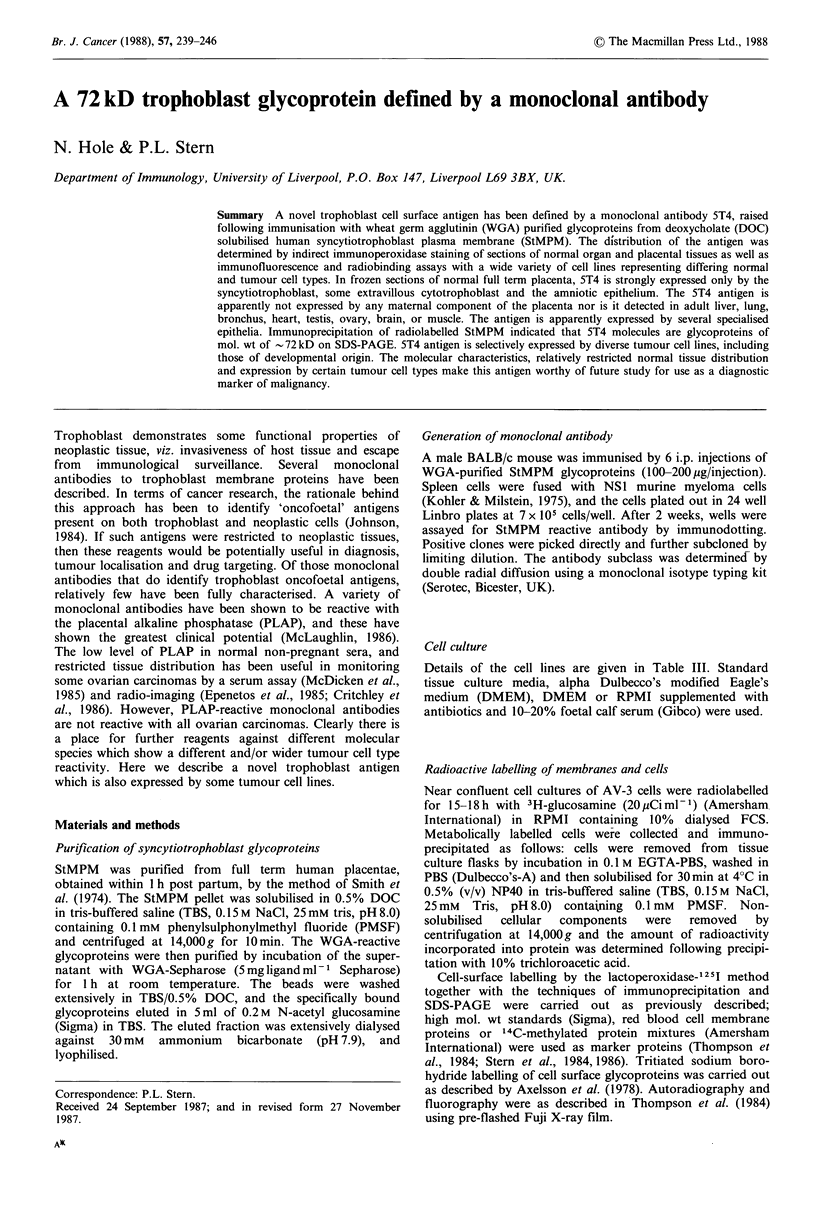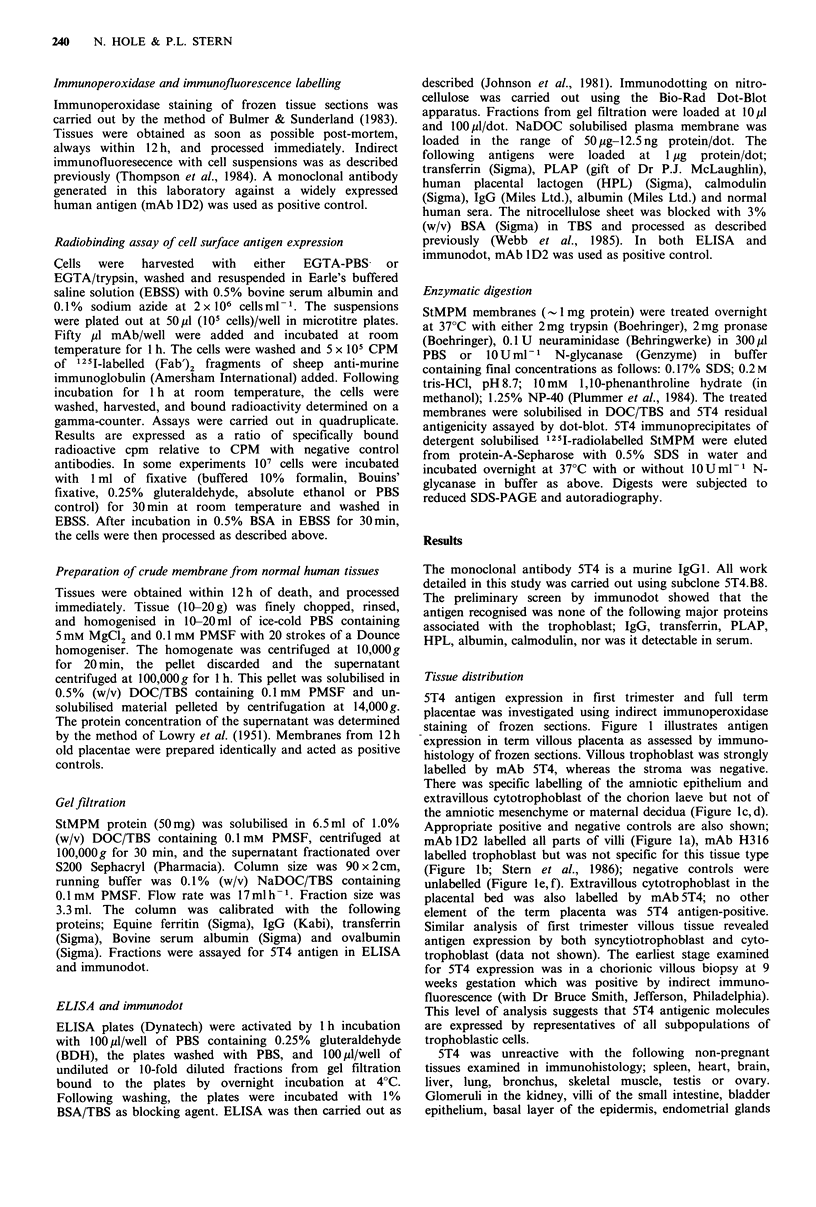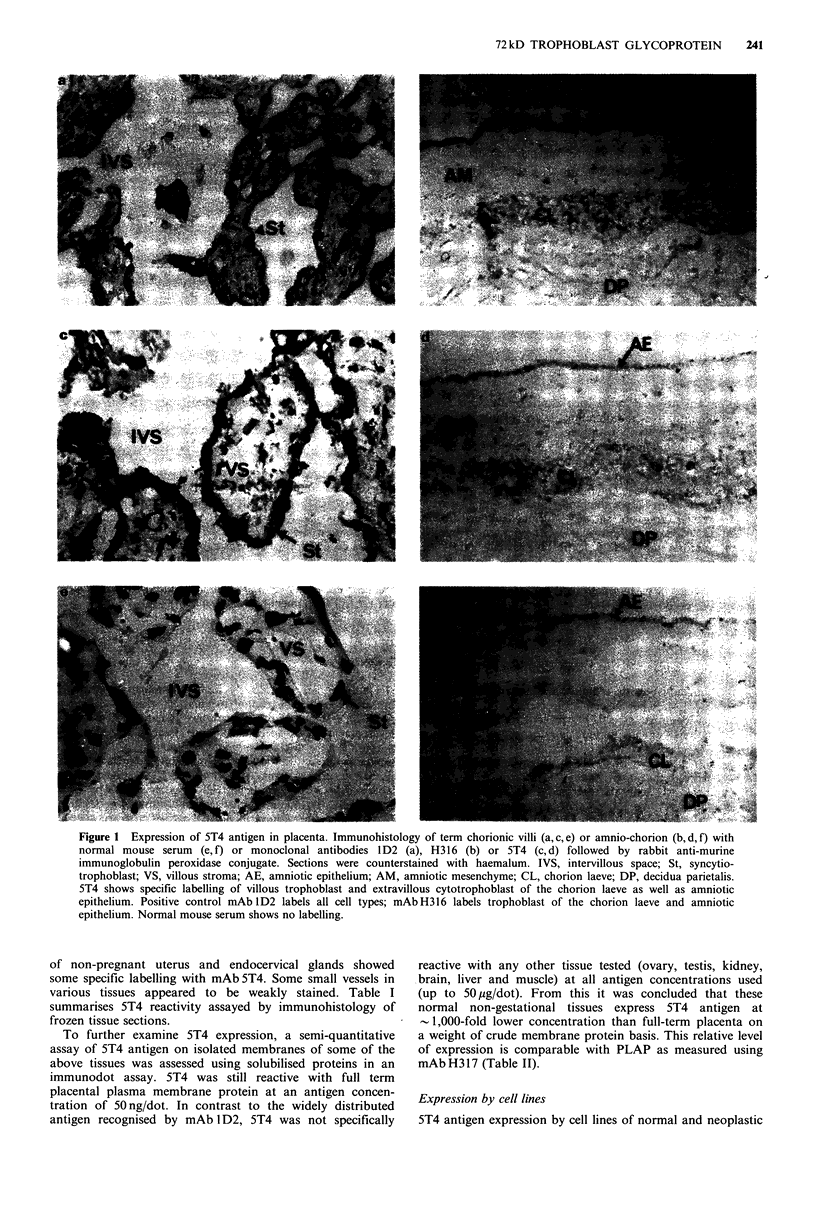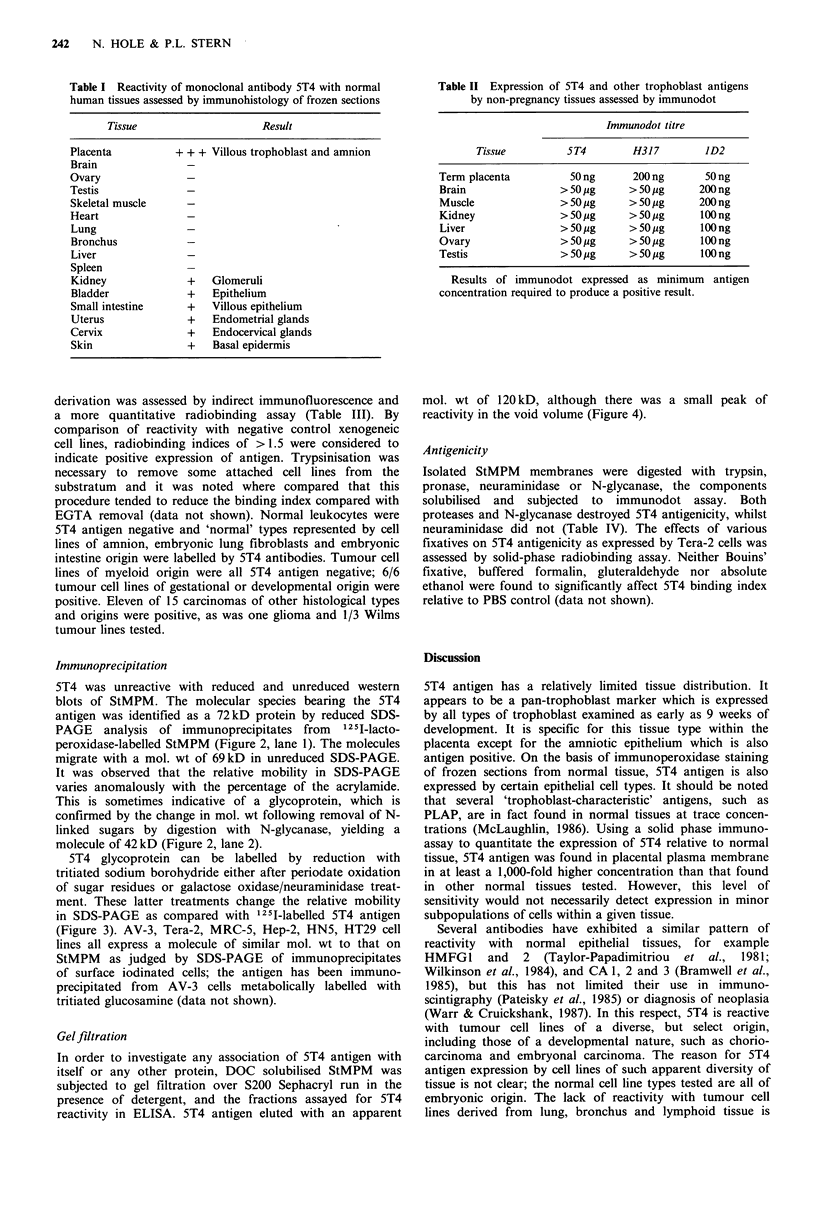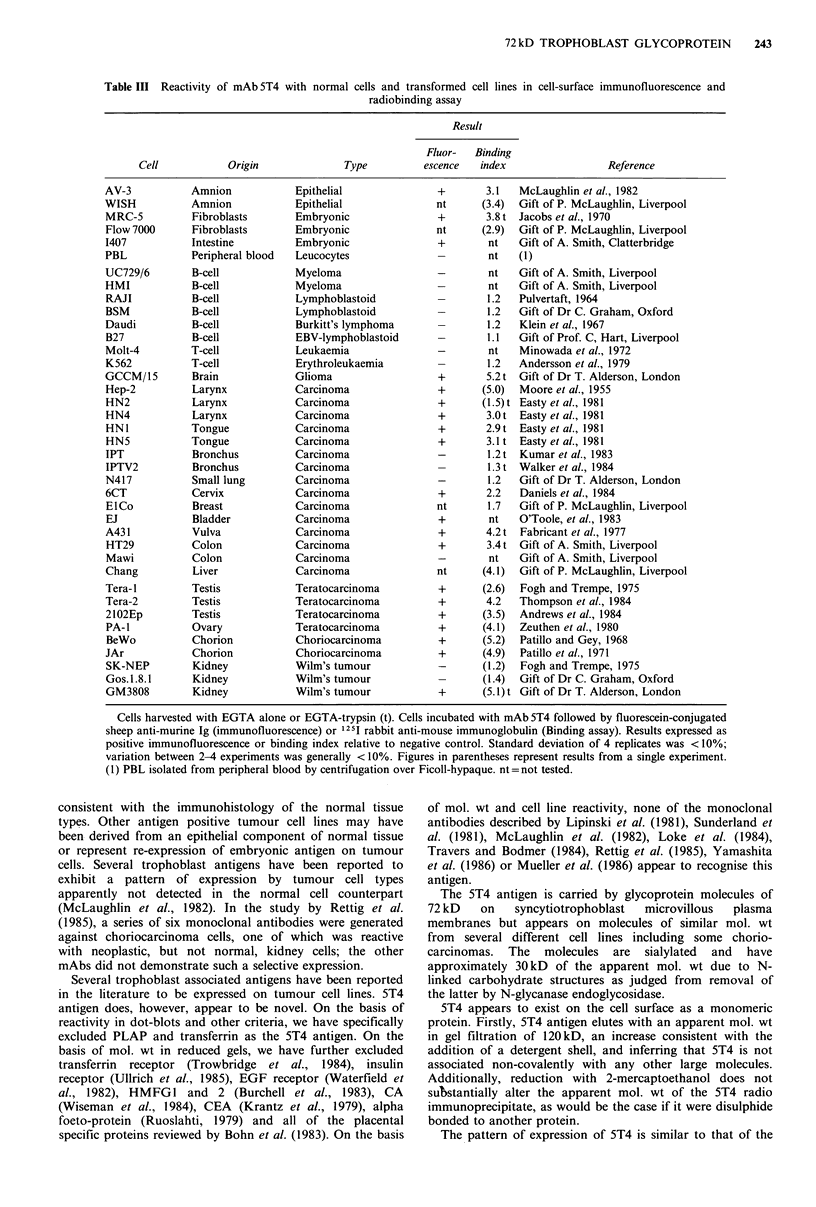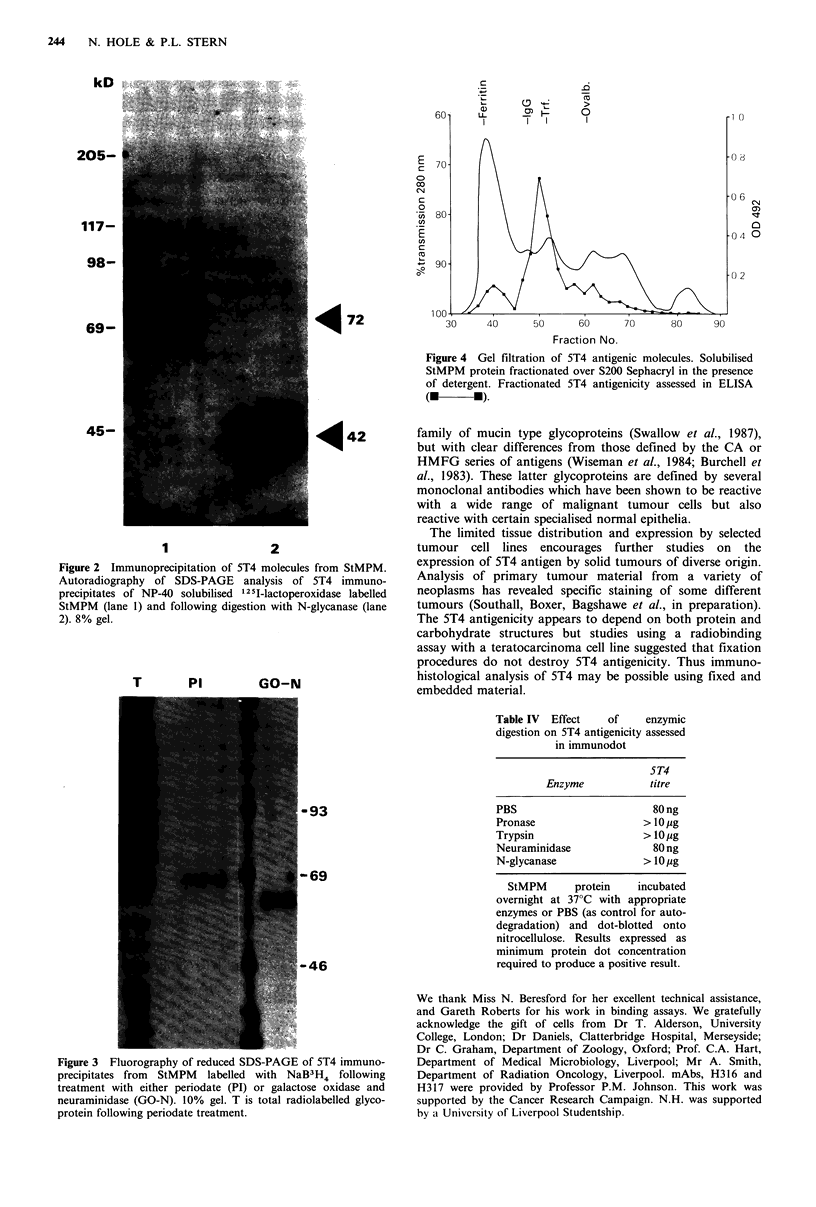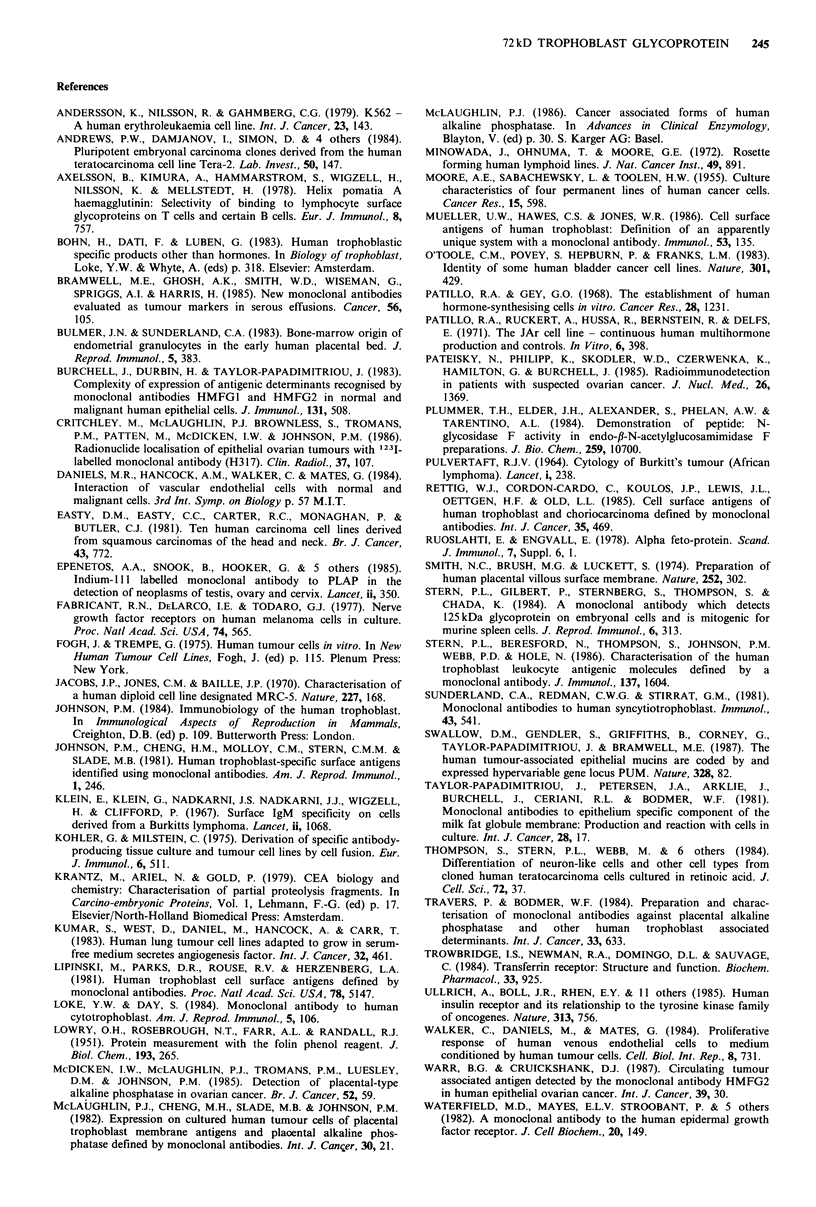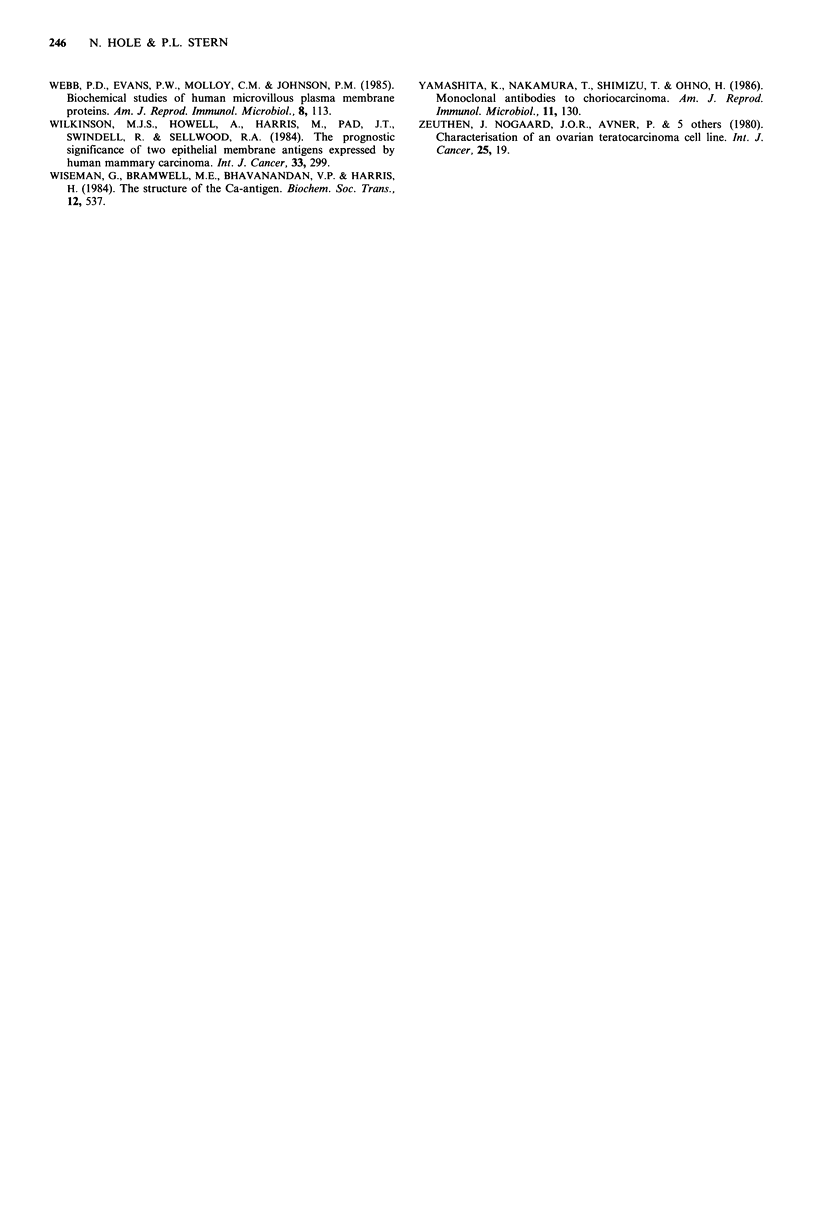# A 72 kD trophoblast glycoprotein defined by a monoclonal antibody.

**DOI:** 10.1038/bjc.1988.53

**Published:** 1988-03

**Authors:** N. Hole, P. L. Stern

**Affiliations:** Department of Immunology, University of Liverpool, UK.

## Abstract

**Images:**


*Br. J. Cancer* (1988), **57**, 239–246 © The Macmillan Press Ltd., 1988

### A 72 kD trophoblast glycoprotein defined by a monoclonal antibody

N. Hole & P.L. Stern

*Department of Immunology, University of Liverpool, P.O. Box 147, Liverpool L69 3BX, UK.*

**Summary** A novel trophoblast cell surface antigen has been defined by a monoclonal antibody 5T4, raised following immunisation with wheat germ agglutinin (WGA) purified glycoproteins from deoxycholate (DOC) solubilised human syncytiotrophoblast plasma membrane (StMPM). The distribution of the antigen was determined by indirect immunoperoxidase staining of sections of normal organ and placental tissues as well as immunofluorescence and radiobinding assays with a wide variety of cell lines representing differing normal and tumour cell types. In frozen sections of normal full term placenta, 5T4 is strongly expressed only by the syncytiotrophoblast, some extravillous cytotrophoblast and the amniotic epithelium. The 5T4 antigen is apparently not expressed by any maternal component of the placenta nor is it detected in adult liver, lung, bronchus, heart, testis, ovary, brain, or muscle. The antigen is apparently expressed by several specialised epithelia. Immunoprecipitation of radiolabelled StMPM indicated that 5T4 molecules are glycoproteins of mol. wt of ~72 kD on SDS-PAGE. 5T4 antigen is selectively expressed by diverse tumour cell lines, including those of developmental origin. The molecular characteristics, relatively restricted normal tissue distribution and expression by certain tumour cell types make this antigen worthy of future study for use as a diagnostic marker of malignancy.

Trophoblast demonstrates some functional properties of neoplastic tissue, *viz.* invasiveness of host tissue and escape from immunological surveillance. Several monoclonal antibodies to trophoblast membrane proteins have been described. In terms of cancer research, the rationale behind this approach has been to identify 'oncofoetal' antigens present on both trophoblast and neoplastic cells (Johnson, 1984). If such antigens were restricted to neoplastic tissues, then these reagents would be potentially useful in diagnosis, tumour localisation and drug targeting. Of those monoclonal antibodies that do identify trophoblast oncofoetal antigens, relatively few have been fully characterised. A variety of monoclonal antibodies have been shown to be reactive with the placental alkaline phosphatase (PLAP), and these have shown the greatest clinical potential (McLaughlin, 1986). The low level of PLAP in normal non-pregnant sera, and restricted tissue distribution has been useful in monitoring some ovarian carcinomas by a serum assay (McDicken *et al.*, 1985) and radio-imaging (Epenetos *et al.*, 1985; Critchley *et al.*, 1986). However, PLAP-reactive monoclonal antibodies are not reactive with all ovarian carcinomas. Clearly there is a place for further reagents against different molecular species which show a different and/or wider tumour cell type reactivity. Here we describe a novel trophoblast antigen which is also expressed by some tumour cell lines.

### Materials and methods

### *Purification of syncytiotrophoblast glycoproteins*

StMPM was purified from full term human placentae, obtained within 1 h post partum, by the method of Smith *et al.* (1974). The StMPM pellet was solubilised in 0.5% DOC in tris-buffered saline (TBS, 0.15 M NaCl, 25 mM tris, pH 8.0) containing 0.1 mM phenylsulphonylmethyl fluoride (PMSF) and centrifuged at 14,000 *g* for 10 min. The WGA-reactive glycoproteins were then purified by incubation of the supernatant with WGA-Sepharose (5 mg ligand $ml^{-1}$ Sepharose) for 1 h at room temperature. The beads were washed extensively in TBS/0.5% DOC, and the specifically bound glycoproteins eluted in 5 ml of 0.2 M N-acetyl glucosamine (Sigma) in TBS. The eluted fraction was extensively dialysed against 30 mM ammonium bicarbonate (pH 7.9), and lyophilised.

### *Generation of monoclonal antibody*

A male BALB/c mouse was immunised by 6 i.p. injections of WGA-purified StMPM glycoproteins (100–200 μg/injection). Spleen cells were fused with NS1 murine myeloma cells (Kohler & Milstein, 1975), and the cells plated out in 24 well Linbro plates at $7 \times 10^5$ cells/well. After 2 weeks, wells were assayed for StMPM reactive antibody by immunodotting. Positive clones were picked directly and further subcloned by limiting dilution. The antibody subclass was determined by double radial diffusion using a monoclonal isotype typing kit (Serotec, Bicester, UK).

### *Cell culture*

Details of the cell lines are given in Table III. Standard tissue culture media, alpha Dulbecco's modified Eagle's medium (DMEM), DMEM or RPMI supplemented with antibiotics and 10–20% foetal calf serum (Gibco) were used.

### *Radioactive labelling of membranes and cells*

Near confluent cell cultures of AV-3 cells were radiolabelled for 15–18 h with $^{3}H$-glucosamine (20 μCi $ml^{-1}$) (Amersham International) in RPMI containing 10% dialysed FCS. Metabolically labelled cells were collected and immunoprecipitated as follows: cells were removed from tissue culture flasks by incubation in 0.1 M EGTA-PBS, washed in PBS (Dulbecco's-A) and then solubilised for 30 min at 4°C in 0.5% (v/v) NP40 in tris-buffered saline (TBS, 0.15 M NaCl, 25 mM Tris, pH 8.0) containing 0.1 mM PMSF. Non-solubilised cellular components were removed by centrifugation at 14,000 *g* and the amount of radioactivity incorporated into protein was determined following precipitation with 10% trichloroacetic acid.

Cell-surface labelling by the lactoperoxidase-$^{125}I$ method together with the techniques of immunoprecipitation and SDS-PAGE were carried out as previously described; high mol. wt standards (Sigma), red blood cell membrane proteins or $^{14}C$-methylated protein mixtures (Amersham International) were used as marker proteins (Thompson *et al.*, 1984; Stern *et al.*, 1984, 1986). Tritiated sodium borohydride labelling of cell surface glycoproteins was carried out as described by Axelsson *et al.* (1978). Autoradiography and fluorography were as described in Thompson *et al.* (1984) using pre-flashed Fuji X-ray film.

Correspondence: P.L. Stern.
Received 24 September 1987; and in revised form 27 November 1987.

*Immunoperoxidase and immunofluorescence labelling*

Immunoperoxidase staining of frozen tissue sections was carried out by the method of Bulmer & Sunderland (1983). Tissues were obtained as soon as possible post-mortem, always within 12 h, and processed immediately. Indirect immunofluoresecence with cell suspensions was as described previously (Thompson *et al.*, 1984). A monoclonal antibody generated in this laboratory against a widely expressed human antigen (mAb 1D2) was used as positive control.

*Radiobinding assay of cell surface antigen expression*

Cells were harvested with either EGTA-PBS or EGTA/trypsin, washed and resuspended in Earle's buffered saline solution (EBSS) with 0.5% bovine serum albumin and 0.1% sodium azide at $2 \times 10^6$ cells ml$^{-1}$. The suspensions were plated out at 50 $\mu$l ($10^5$ cells)/well in microtitre plates. Fifty $\mu$l mAb/well were added and incubated at room temperature for 1 h. The cells were washed and $5 \times 10^5$ CPM of $^{125}$I-labelled (Fab')$_2$ fragments of sheep anti-murine immunoglobulin (Amersham International) added. Following incubation for 1 h at room temperature, the cells were washed, harvested, and bound radioactivity determined on a gamma-counter. Assays were carried out in quadruplicate. Results are expressed as a ratio of specifically bound radioactive cpm relative to CPM with negative control antibodies. In some experiments $10^7$ cells were incubated with 1 ml of fixative (buffered 10% formalin, Bouins' fixative, 0.25% gluteraldehyde, absolute ethanol or PBS control) for 30 min at room temperature and washed in EBSS. After incubation in 0.5% BSA in EBSS for 30 min, the cells were then processed as described above.

*Preparation of crude membrane from normal human tissues*

Tissues were obtained within 12 h of death, and processed immediately. Tissue (10–20 g) was finely chopped, rinsed, and homogenised in 10–20 ml of ice-cold PBS containing 5 mM $MgCl_2$ and 0.1 mM PMSF with 20 strokes of a Dounce homogeniser. The homogenate was centrifuged at 10,000 *g* for 20 min, the pellet discarded and the supernatant centrifuged at 100,000 *g* for 1 h. This pellet was solubilised in 0.5% (w/v) DOC/TBS containing 0.1 mM PMSF and unsolubilised material pelleted by centrifugation at 14,000 *g*. The protein concentration of the supernatant was determined by the method of Lowry *et al.* (1951). Membranes from 12 h old placentae were prepared identically and acted as positive controls.

*Gel filtration*

StMPM protein (50 mg) was solubilised in 6.5 ml of 1.0% (w/v) DOC/TBS containing 0.1 mM PMSF, centrifuged at 100,000 *g* for 30 min, and the supernatant fractionated over S200 Sephacryl (Pharmacia). Column size was 90 × 2 cm, running buffer was 0.1% (w/v) NaDOC/TBS containing 0.1 mM PMSF. Flow rate was 17 ml h$^{-1}$. Fraction size was 3.3 ml. The column was calibrated with the following proteins; Equine ferritin (Sigma), IgG (Kabi), transferrin (Sigma), Bovine serum albumin (Sigma) and ovalbumin (Sigma). Fractions were assayed for 5T4 antigen in ELISA and immunodot.

*ELISA and immunodot*

ELISA plates (Dynatech) were activated by 1 h incubation with 100 $\mu$l/well of PBS containing 0.25% gluteraldehyde (BDH), the plates washed with PBS, and 100 $\mu$l/well of undiluted or 10-fold diluted fractions from gel filtration bound to the plates by overnight incubation at 4°C. Following washing, the plates were incubated with 1% BSA/TBS as blocking agent. ELISA was then carried out as described (Johnson *et al.*, 1981). Immunodotting on nitrocellulose was carried out using the Bio-Rad Dot-Blot apparatus. Fractions from gel filtration were loaded at 10 $\mu$l and 100 $\mu$l/dot. NaDOC solubilised plasma membrane was loaded in the range of 50 $\mu$g–12.5 ng protein/dot. The following antigens were loaded at 1 $\mu$g protein/dot; transferrin (Sigma), PLAP (gift of Dr P.J. McLaughlin), human placental lactogen (HPL) (Sigma), calmodulin (Sigma), IgG (Miles Ltd.), albumin (Miles Ltd.) and normal human sera. The nitrocellulose sheet was blocked with 3% (w/v) BSA (Sigma) in TBS and processed as described previously (Webb *et al.*, 1985). In both ELISA and immunodot, mAb 1D2 was used as positive control.

*Enzymatic digestion*

StMPM membranes (~1 mg protein) were treated overnight at 37°C with either 2 mg trypsin (Boehringer), 2 mg pronase (Boehringer), 0.1 U neuraminidase (Behringwerke) in 300 $\mu$l PBS or 10 U ml$^{-1}$ N-glycanase (Genzyme) in buffer containing final concentrations as follows: 0.17% SDS; 0.2 M tris-HCl, pH 8.7; 10 mM 1,10-phenanthroline hydrate (in methanol); 1.25% NP-40 (Plummer *et al.*, 1984). The treated membranes were solubilised in DOC/TBS and 5T4 residual antigenicity assayed by dot-blot. 5T4 immunoprecipitates of detergent solubilised $^{125}$I-radiolabelled StMPM were eluted from protein-A-Sepharose with 0.5% SDS in water and incubated overnight at 37°C with or without 10 U ml$^{-1}$ N-glycanase in buffer as above. Digests were subjected to reduced SDS-PAGE and autoradiography.

**Results**

The monoclonal antibody 5T4 is a murine IgG1. All work detailed in this study was carried out using subclone 5T4.B8. The preliminary screen by immunodot showed that the antigen recognised was none of the following major proteins associated with the trophoblast; IgG, transferrin, PLAP, HPL, albumin, calmodulin, nor was it detectable in serum.

*Tissue distribution*

5T4 antigen expression in first trimester and full term placentae was investigated using indirect immunoperoxidase staining of frozen sections. Figure 1 illustrates antigen expression in term villous placenta as assessed by immunohistology of frozen sections. Villous trophoblast was strongly labelled by mAb 5T4, whereas the stroma was negative. There was specific labelling of the amniotic epithelium and extravillous cytotrophoblast of the chorion laeve but not of the amniotic mesenchyme or maternal decidua (Figure 1c, d). Appropriate positive and negative controls are also shown; mAb 1D2 labelled all parts of villi (Figure 1a), mAb H316 labelled trophoblast but was not specific for this tissue type (Figure 1b; Stern *et al.*, 1986); negative controls were unlabelled (Figure 1e, f). Extravillous cytotrophoblast in the placental bed was also labelled by mAb 5T4; no other element of the term placenta was 5T4 antigen-positive. Similar analysis of first trimester villous tissue revealed antigen expression by both syncytiotrophoblast and cytotrophoblast (data not shown). The earliest stage examined for 5T4 expression was in a chorionic villous biopsy at 9 weeks gestation which was positive by indirect immunofluorescence (with Dr Bruce Smith, Jefferson, Philadelphia). This level of analysis suggests that 5T4 antigenic molecules are expressed by representatives of all subpopulations of trophoblastic cells.

5T4 was unreactive with the following non-pregnant tissues examined in immunohistology; spleen, heart, brain, liver, lung, bronchus, skeletal muscle, testis or ovary. Glomeruli in the kidney, villi of the small intestine, bladder epithelium, basal layer of the epidermis, endometrial glands

**Figure 1** Expression of 5T4 antigen in placenta. Immunohistology of term chorionic villi (a, c, e) or amnio-chorion (b, d, f) with normal mouse serum (e, f) or monoclonal antibodies 1D2 (a), H316 (b) or 5T4 (c, d) followed by rabbit anti-murine immunoglobulin peroxidase conjugate. Sections were counterstained with haemalum. IVS, intervillous space; St, syncytio-trophoblast; VS, villous stroma; AE, amniotic epithelium; AM, amniotic mesenchyme; CL, chorion laeve; DP, decidua parietalis. 5T4 shows specific labelling of villous trophoblast and extravillous cytotrophoblast of the chorion laeve as well as amniotic epithelium. Positive control mAb 1D2 labels all cell types; mAb H316 labels trophoblast of the chorion laeve and amniotic epithelium. Normal mouse serum shows no labelling.

of non-pregnant uterus and endocervical glands showed some specific labelling with mAb 5T4. Some small vessels in various tissues appeared to be weakly stained. Table I summarises 5T4 reactivity assayed by immunohistology of frozen tissue sections.

To further examine 5T4 expression, a semi-quantitative assay of 5T4 antigen on isolated membranes of some of the above tissues was assessed using solubilised proteins in an immunodot assay. 5T4 was still reactive with full term placental plasma membrane protein at an antigen concentration of 50 ng/dot. In contrast to the widely distributed antigen recognised by mAb 1D2, 5T4 was not specifically reactive with any other tissue tested (ovary, testis, kidney, brain, liver and muscle) at all antigen concentrations used (up to 50 $\mu$g/dot). From this it was concluded that these normal non-gestational tissues express 5T4 antigen at $\sim$1,000-fold lower concentration than full-term placenta on a weight of crude membrane protein basis. This relative level of expression is comparable with PLAP as measured using mAb H317 (Table II).

*Expression by cell lines*

5T4 antigen expression by cell lines of normal and neoplastic

**Table I** Reactivity of monoclonal antibody 5T4 with normal human tissues assessed by immunohistology of frozen sections

| *Tissue* | *Result* | |
|---|---|---|
| Placenta | + + + | Villous trophoblast and amnion |
| Brain | − | |
| Ovary | − | |
| Testis | − | |
| Skeletal muscle | − | |
| Heart | − | |
| Lung | − | |
| Bronchus | − | |
| Liver | − | |
| Spleen | − | |
| Kidney | + | Glomeruli |
| Bladder | + | Epithelium |
| Small intestine | + | Villous epithelium |
| Uterus | + | Endometrial glands |
| Cervix | + | Endocervical glands |
| Skin | + | Basal epidermis |

**Table II** Expression of 5T4 and other trophoblast antigens by non-pregnancy tissues assessed by immunodot

| | *Immunodot titre* | | |
|---|---|---|---|
| *Tissue* | *5T4* | *H317* | *1D2* |
| Term placenta | 50 ng | 200 ng | 50 ng |
| Brain | >50 μg | >50 μg | 200 ng |
| Muscle | >50 μg | >50 μg | 200 ng |
| Kidney | >50 μg | >50 μg | 100 ng |
| Liver | >50 μg | >50 μg | 100 ng |
| Ovary | >50 μg | >50 μg | 100 ng |
| Testis | >50 μg | >50 μg | 100 ng |

Results of immunodot expressed as minimum antigen concentration required to produce a positive result.

derivation was assessed by indirect immunofluorescence and a more quantitative radiobinding assay (Table III). By comparison of reactivity with negative control xenogeneic cell lines, radiobinding indices of >1.5 were considered to indicate positive expression of antigen. Trypsinisation was necessary to remove some attached cell lines from the substratum and it was noted where compared that this procedure tended to reduce the binding index compared with EGTA removal (data not shown). Normal leukocytes were 5T4 antigen negative and 'normal' types represented by cell lines of amnion, embryonic lung fibroblasts and embryonic intestine origin were labelled by 5T4 antibodies. Tumour cell lines of myeloid origin were all 5T4 antigen negative; 6/6 tumour cell lines of gestational or developmental origin were positive. Eleven of 15 carcinomas of other histological types and origins were positive, as was one glioma and 1/3 Wilms tumour lines tested.

### *Immunoprecipitation*

5T4 was unreactive with reduced and unreduced western blots of StMPM. The molecular species bearing the 5T4 antigen was identified as a 72 kD protein by reduced SDS-PAGE analysis of immunoprecipitates from $^{125}$I-lactoperoxidase-labelled StMPM (Figure 2, lane 1). The molecules migrate with a mol. wt of 69 kD in unreduced SDS-PAGE. It was observed that the relative mobility in SDS-PAGE varies anomalously with the percentage of the acrylamide. This is sometimes indicative of a glycoprotein, which is confirmed by the change in mol. wt following removal of N-linked sugars by digestion with N-glycanase, yielding a molecule of 42 kD (Figure 2, lane 2).

5T4 glycoprotein can be labelled by reduction with tritiated sodium borohydride either after periodate oxidation of sugar residues or galactose oxidase/neuraminidase treatment. These latter treatments change the relative mobility in SDS-PAGE as compared with $^{125}$I-labelled 5T4 antigen (Figure 3). AV-3, Tera-2, MRC-5, Hep-2, HN5, HT29 cell lines all express a molecule of similar mol. wt to that on StMPM as judged by SDS-PAGE of immunoprecipitates of surface iodinated cells; the antigen has been immunoprecipitated from AV-3 cells metabolically labelled with tritiated glucosamine (data not shown).

### *Gel filtration*

In order to investigate any association of 5T4 antigen with itself or any other protein, DOC solubilised StMPM was subjected to gel filtration over S200 Sephacryl run in the presence of detergent, and the fractions assayed for 5T4 reactivity in ELISA. 5T4 antigen eluted with an apparent mol. wt of 120 kD, although there was a small peak of reactivity in the void volume (Figure 4).

### *Antigenicity*

Isolated StMPM membranes were digested with trypsin, pronase, neuraminidase or N-glycanase, the components solubilised and subjected to immunodot assay. Both proteases and N-glycanase destroyed 5T4 antigenicity, whilst neuraminidase did not (Table IV). The effects of various fixatives on 5T4 antigenicity as expressed by Tera-2 cells was assessed by solid-phase radiobinding assay. Neither Bouins' fixative, buffered formalin, gluteraldehyde nor absolute ethanol were found to significantly affect 5T4 binding index relative to PBS control (data not shown).

### Discussion

5T4 antigen has a relatively limited tissue distribution. It appears to be a pan-trophoblast marker which is expressed by all types of trophoblast examined as early as 9 weeks of development. It is specific for this tissue type within the placenta except for the amniotic epithelium which is also antigen positive. On the basis of immunoperoxidase staining of frozen sections from normal tissue, 5T4 antigen is also expressed by certain epithelial cell types. It should be noted that several 'trophoblast-characteristic' antigens, such as PLAP, are in fact found in normal tissues at trace concentrations (McLaughlin, 1986). Using a solid phase immunoassay to quantitate the expression of 5T4 relative to normal tissue, 5T4 antigen was found in placental plasma membrane in at least a 1,000-fold higher concentration than that found in other normal tissues tested. However, this level of sensitivity would not necessarily detect expression in minor subpopulations of cells within a given tissue.

Several antibodies have exhibited a similar pattern of reactivity with normal epithelial tissues, for example HMFG1 and 2 (Taylor-Papadimitriou *et al.*, 1981; Wilkinson *et al.*, 1984), and CA 1, 2 and 3 (Bramwell *et al.*, 1985), but this has not limited their use in immunoscintigraphy (Pateisky *et al.*, 1985) or diagnosis of neoplasia (Warr & Cruickshank, 1987). In this respect, 5T4 is reactive with tumour cell lines of a diverse, but select origin, including those of a developmental nature, such as choriocarcinoma and embryonal carcinoma. The reason for 5T4 antigen expression by cell lines of such apparent diversity of tissue is not clear; the normal cell line types tested are all of embryonic origin. The lack of reactivity with tumour cell lines derived from lung, bronchus and lymphoid tissue is

**Table III** Reactivity of mAb 5T4 with normal cells and transformed cell lines in cell-surface immunofluorescence and radiobinding assay

| Cell | Origin | Type | Result: Fluorescence | Result: Binding index | Reference |
|---|---|---|---|---|---|
| AV-3 | Amnion | Epithelial | + | 3.1 | McLaughlin *et al.*, 1982 |
| WISH | Amnion | Epithelial | nt | (3.4) | Gift of P. McLaughlin, Liverpool |
| MRC-5 | Fibroblasts | Embryonic | + | 3.8 t | Jacobs *et al.*, 1970 |
| Flow 7000 | Fibroblasts | Embryonic | nt | (2.9) | Gift of P. McLaughlin, Liverpool |
| I407 | Intestine | Embryonic | + | nt | Gift of A. Smith, Clatterbridge |
| PBL | Peripheral blood | Leucocytes | – | nt | (1) |
| UC729/6 | B-cell | Myeloma | – | nt | Gift of A. Smith, Liverpool |
| HMI | B-cell | Myeloma | – | nt | Gift of A. Smith, Liverpool |
| RAJI | B-cell | Lymphoblastoid | – | 1.2 | Pulvertaft, 1964 |
| BSM | B-cell | Lymphoblastoid | – | 1.2 | Gift of Dr C. Graham, Oxford |
| Daudi | B-cell | Burkitt's lymphoma | – | 1.2 | Klein *et al.*, 1967 |
| B27 | B-cell | EBV-lymphoblastoid | – | 1.1 | Gift of Prof. C, Hart, Liverpool |
| Molt-4 | T-cell | Leukaemia | – | nt | Minowada *et al.*, 1972 |
| K562 | T-cell | Erythroleukaemia | – | 1.2 | Andersson *et al.*, 1979 |
| GCCM/15 | Brain | Glioma | + | 5.2 t | Gift of Dr T. Alderson, London |
| Hep-2 | Larynx | Carcinoma | + | (5.0) | Moore *et al.*, 1955 |
| HN2 | Larynx | Carcinoma | + | (1.5) t | Easty *et al.*, 1981 |
| HN4 | Larynx | Carcinoma | + | 3.0 t | Easty *et al.*, 1981 |
| HN1 | Tongue | Carcinoma | + | 2.9 t | Easty *et al.*, 1981 |
| HN5 | Tongue | Carcinoma | + | 3.1 t | Easty *et al.*, 1981 |
| IPT | Bronchus | Carcinoma | – | 1.2 t | Kumar *et al.*, 1983 |
| IPTV2 | Bronchus | Carcinoma | – | 1.3 t | Walker *et al.*, 1984 |
| N417 | Small lung | Carcinoma | – | 1.2 | Gift of Dr T. Alderson, London |
| 6CT | Cervix | Carcinoma | + | 2.2 | Daniels *et al.*, 1984 |
| E1Co | Breast | Carcinoma | nt | 1.7 | Gift of P. McLaughlin, Liverpool |
| EJ | Bladder | Carcinoma | + | nt | O'Toole, *et al.*, 1983 |
| A431 | Vulva | Carcinoma | + | 4.2 t | Fabricant *et al.*, 1977 |
| HT29 | Colon | Carcinoma | + | 3.4 t | Gift of A. Smith, Liverpool |
| Mawi | Colon | Carcinoma | – | nt | Gift of A. Smith, Liverpool |
| Chang | Liver | Carcinoma | nt | (4.1) | Gift of P. McLaughlin, Liverpool |
| Tera-1 | Testis | Teratocarcinoma | + | (2.6) | Fogh and Trempe, 1975 |
| Tera-2 | Testis | Teratocarcinoma | + | 4.2 | Thompson *et al.*, 1984 |
| 2102Ep | Testis | Teratocarcinoma | + | (3.5) | Andrews *et al.*, 1984 |
| PA-1 | Ovary | Teratocarcinoma | + | (4.1) | Zeuthen *et al.*, 1980 |
| BeWo | Chorion | Choriocarcinoma | + | (5.2) | Patillo and Gey, 1968 |
| JAr | Chorion | Choriocarcinoma | + | (4.9) | Patillo *et al.*, 1971 |
| SK-NEP | Kidney | Wilm's tumour | – | (1.2) | Fogh and Trempe, 1975 |
| Gos.1.8.1 | Kidney | Wilm's tumour | – | (1.4) | Gift of Dr C. Graham, Oxford |
| GM3808 | Kidney | Wilm's tumour | + | (5.1) t | Gift of Dr T. Alderson, London |

Cells harvested with EGTA alone or EGTA-trypsin (t). Cells incubated with mAb 5T4 followed by fluorescein-conjugated sheep anti-murine Ig (immunofluorescence) or $^{125}I$ rabbit anti-mouse immunoglobulin (Binding assay). Results expressed as positive immunofluorescence or binding index relative to negative control. Standard deviation of 4 replicates was <10%; variation between 2–4 experiments was generally <10%. Figures in parentheses represent results from a single experiment. (1) PBL isolated from peripheral blood by centrifugation over Ficoll-hypaque. nt = not tested.

consistent with the immunohistology of the normal tissue types. Other antigen positive tumour cell lines may have been derived from an epithelial component of normal tissue or represent re-expression of embryonic antigen on tumour cells. Several trophoblast antigens have been reported to exhibit a pattern of expression by tumour cell types apparently not detected in the normal cell counterpart (McLaughlin *et al.*, 1982). In the study by Rettig *et al.* (1985), a series of six monoclonal antibodies were generated against choriocarcinoma cells, one of which was reactive with neoplastic, but not normal, kidney cells; the other mAbs did not demonstrate such a selective expression.

Several trophoblast associated antigens have been reported in the literature to be expressed on tumour cell lines. 5T4 antigen does, however, appear to be novel. On the basis of reactivity in dot-blots and other criteria, we have specifically excluded PLAP and transferrin as the 5T4 antigen. On the basis of mol. wt in reduced gels, we have further excluded transferrin receptor (Trowbridge *et al.*, 1984), insulin receptor (Ullrich *et al.*, 1985), EGF receptor (Waterfield *et al.*, 1982), HMFG1 and 2 (Burchell *et al.*, 1983), CA (Wiseman *et al.*, 1984), CEA (Krantz *et al.*, 1979), alpha foeto-protein (Ruoslahti, 1979) and all of the placental specific proteins reviewed by Bohn *et al.* (1983). On the basis of mol. wt and cell line reactivity, none of the monoclonal antibodies described by Lipinski *et al.* (1981), Sunderland *et al.* (1981), McLaughlin *et al.* (1982), Loke *et al.* (1984), Travers and Bodmer (1984), Rettig *et al.* (1985), Yamashita *et al.* (1986) or Mueller *et al.* (1986) appear to recognise this antigen.

The 5T4 antigen is carried by glycoprotein molecules of 72 kD on syncytiotrophoblast microvillous plasma membranes but appears on molecules of similar mol. wt from several different cell lines including some choriocarcinomas. The molecules are sialylated and have approximately 30 kD of the apparent mol. wt due to N-linked carbohydrate structures as judged from removal of the latter by N-glycanase endoglycosidase.

5T4 appears to exist on the cell surface as a monomeric protein. Firstly, 5T4 antigen elutes with an apparent mol. wt in gel filtration of 120 kD, an increase consistent with the addition of a detergent shell, and inferring that 5T4 is not associated non-covalently with any other large molecules. Additionally, reduction with 2-mercaptoethanol does not substantially alter the apparent mol. wt of the 5T4 radio immunoprecipitate, as would be the case if it were disulphide bonded to another protein.

The pattern of expression of 5T4 is similar to that of the

**Figure 2** Immunoprecipitation of 5T4 molecules from StMPM. Autoradiography of SDS-PAGE analysis of 5T4 immunoprecipitates of NP-40 solubilised $^{125}$I-lactoperoxidase labelled StMPM (lane 1) and following digestion with N-glycanase (lane 2). 8% gel.

**Figure 3** Fluorography of reduced SDS-PAGE of 5T4 immunoprecipitates from StMPM labelled with $NaB^3H_4$ following treatment with either periodate (PI) or galactose oxidase and neuraminidase (GO-N). 10% gel. T is total radiolabelled glycoprotein following periodate treatment.

**Figure 4** Gel filtration of 5T4 antigenic molecules. Solubilised StMPM protein fractionated over S200 Sephacryl in the presence of detergent. Fractionated 5T4 antigenicity assessed in ELISA (■——■).

family of mucin type glycoproteins (Swallow *et al.*, 1987), but with clear differences from those defined by the CA or HMFG series of antigens (Wiseman *et al.*, 1984; Burchell *et al.*, 1983). These latter glycoproteins are defined by several monoclonal antibodies which have been shown to be reactive with a wide range of malignant tumour cells but also reactive with certain specialised normal epithelia.

The limited tissue distribution and expression by selected tumour cell lines encourages further studies on the expression of 5T4 antigen by solid tumours of diverse origin. Analysis of primary tumour material from a variety of neoplasms has revealed specific staining of some different tumours (Southall, Boxer, Bagshawe *et al.*, in preparation). The 5T4 antigenicity appears to depend on both protein and carbohydrate structures but studies using a radiobinding assay with a teratocarcinoma cell line suggested that fixation procedures do not destroy 5T4 antigenicity. Thus immunohistological analysis of 5T4 may be possible using fixed and embedded material.

**Table IV** Effect of enzymic digestion on 5T4 antigenicity assessed in immunodot

| *Enzyme* | *5T4 titre* |
|---|---|
| PBS | 80 ng |
| Pronase | >10 μg |
| Trypsin | >10 μg |
| Neuraminidase | 80 ng |
| N-glycanase | >10 μg |

StMPM protein incubated overnight at 37°C with appropriate enzymes or PBS (as control for autodegradation) and dot-blotted onto nitrocellulose. Results expressed as minimum protein dot concentration required to produce a positive result.

We thank Miss N. Beresford for her excellent technical assistance, and Gareth Roberts for his work in binding assays. We gratefully acknowledge the gift of cells from Dr T. Alderson, University College, London; Dr Daniels, Clatterbridge Hospital, Merseyside; Dr C. Graham, Department of Zoology, Oxford; Prof. C.A. Hart, Department of Medical Microbiology, Liverpool; Mr A. Smith, Department of Radiation Oncology, Liverpool. mAbs, H316 and H317 were provided by Professor P.M. Johnson. This work was supported by the Cancer Research Campaign. N.H. was supported by a University of Liverpool Studentship.

### References

ANDERSSON, K., NILSSON, R. & GAHMBERG, C.G. (1979). K562 – A human erythroleukaemia cell line. *Int. J. Cancer*, **23**, 143.

ANDREWS, P.W., DAMJANOV, I., SIMON, D. & 4 others (1984). Pluripotent embryonal carcinoma clones derived from the human teratocarcinoma cell line Tera-2. *Lab. Invest.*, **50**, 147.

AXELSSON, B., KIMURA, A., HAMMARSTROM, S., WIGZELL, H., NILSSON, K. & MELLSTEDT, H. (1978). Helix pomatia A haemagglutinin: Selectivity of binding to lymphocyte surface glycoproteins on T cells and certain B cells. *Eur. J. Immunol.*, **8**, 757.

BOHN, H., DATI, F. & LUBEN, G. (1983). Human trophoblastic specific products other than hormones. In *Biology of trophoblast*, Loke, Y.W. & Whyte, A. (eds) p. 318. Elsevier: Amsterdam.

BRAMWELL, M.E., GHOSH, A.K., SMITH, W.D., WISEMAN, G., SPRIGGS, A.I. & HARRIS, H. (1985). New monoclonal antibodies evaluated as tumour markers in serous effusions. *Cancer*, **56**, 105.

BULMER, J.N. & SUNDERLAND, C.A. (1983). Bone-marrow origin of endometrial granulocytes in the early human placental bed. *J. Reprod. Immunol.*, **5**, 383.

BURCHELL, J., DURBIN, H. & TAYLOR-PAPADIMITRIOU, J. (1983). Complexity of expression of antigenic determinants recognised by monoclonal antibodies HMFG1 and HMFG2 in normal and malignant human epithelial cells. *J. Immunol.*, **131**, 508.

CRITCHLEY, M., McLAUGHLIN, P.J. BROWNLESS, S., TROMANS, P.M., PATTEN, M., McDICKEN, I.W. & JOHNSON, P.M. (1986). Radionuclide localisation of epithelial ovarian tumours with $^{123}$I-labelled monoclonal antibody (H317). *Clin. Radiol.*, **37**, 107.

DANIELS, M.R., HANCOCK, A.M., WALKER, C. & MATES, G. (1984). Interaction of vascular endothelial cells with normal and malignant cells. *3rd Int. Symp. on Biology* p. 57 M.I.T.

EASTY, D.M., EASTY, C.C., CARTER, R.C., MONAGHAN, P. & BUTLER, C.J. (1981). Ten human carcinoma cell lines derived from squamous carcinomas of the head and neck. *Br. J. Cancer*, **43**, 772.

EPENETOS, A.A., SNOOK, B., HOOKER, G. & 5 others (1985). Indium-111 labelled monoclonal antibody to PLAP in the detection of neoplasms of testis, ovary and cervix. *Lancet*, **ii**, 350.

FABRICANT, R.N., DeLARCO, I.E. & TODARO, G.J. (1977). Nerve growth factor receptors on human melanoma cells in culture. *Proc. Natl Acad. Sci. USA*, **74**, 565.

FOGH, J. & TREMPE, G. (1975). Human tumour cells *in vitro*. In *New Human Tumour Cell Lines*, Fogh, J. (ed) p. 115. Plenum Press: New York.

JACOBS, J.P., JONES, C.M. & BAILLE, J.P. (1970). Characterisation of a human diploid cell line designated MRC-5. *Nature*, **227**, 168.

JOHNSON, P.M. (1984). Immunobiology of the human trophoblast. In *Immunological Aspects of Reproduction in Mammals*, Creighton, D.B. (ed) p. 109. Butterworth Press: London.

JOHNSON, P.M., CHENG, H.M., MOLLOY, C.M., STERN, C.M.M. & SLADE, M.B. (1981). Human trophoblast-specific surface antigens identified using monoclonal antibodies. *Am. J. Reprod. Immunol.*, **1**, 246.

KLEIN, E., KLEIN, G., NADKARNI, J.S. NADKARNI, J.J., WIGZELL, H. & CLIFFORD, P. (1967). Surface IgM specificity on cells derived from a Burkitts lymphoma. *Lancet*, **ii**, 1068.

KOHLER, G. & MILSTEIN, C. (1975). Derivation of specific antibody-producing tissue culture and tumour cell lines by cell fusion. *Eur. J. Immunol.*, **6**, 511.

KRANTZ, M., ARIEL, N. & GOLD, P. (1979). CEA biology and chemistry: Characterisation of partial proteolysis fragments. In *Carcino-embryonic Proteins*, Vol. 1, Lehmann, F.-G. (ed) p. 17. Elsevier/North-Holland Biomedical Press: Amsterdam.

KUMAR, S., WEST, D., DANIEL, M., HANCOCK, A. & CARR, T. (1983). Human lung tumour cell lines adapted to grow in serum-free medium secretes angiogenesis factor. *Int. J. Cancer*, **32**, 461.

LIPINSKI, M., PARKS, D.R., ROUSE, R.V. & HERZENBERG, L.A. (1981). Human trophoblast cell surface antigens defined by monoclonal antibodies. *Proc. Natl Acad. Sci. USA*, **78**, 5147.

LOKE, Y.W. & DAY, S. (1984). Monoclonal antibody to human cytotrophoblast. *Am. J. Reprod. Immunol.*, **5**, 106.

LOWRY, O.H., ROSEBROUGH, N.T., FARR, A.L. & RANDALL, R.J. (1951). Protein measurement with the folin phenol reagent. *J. Biol. Chem.*, **193**, 265.

McDICKEN, I.W., McLAUGHLIN, P.J., TROMANS, P.M., LUESLEY, D.M. & JOHNSON, P.M. (1985). Detection of placental-type alkaline phosphatase in ovarian cancer. *Br. J. Cancer*, **52**, 59.

McLAUGHLIN, P.J., CHENG, M.H., SLADE, M.B. & JOHNSON, P.M. (1982). Expression on cultured human tumour cells of placental trophoblast membrane antigens and placental alkaline phosphatase defined by monoclonal antibodies. *Int. J. Cancer*, **30**, 21.

McLAUGHLIN, P.J. (1986). Cancer associated forms of human alkaline phosphatase. In *Advances in Clinical Enzymology*, Blayton, V. (ed) p. 30. S. Karger AG: Basel.

MINOWADA, J., OHNUMA, T. & MOORE, G.E. (1972). Rosette forming human lymphoid lines. *J. Nat. Cancer Inst.*, **49**, 891.

MOORE, A.E., SABACHEWSKY, L. & TOOLEN, H.W. (1955). Culture characteristics of four permanent lines of human cancer cells. *Cancer Res.*, **15**, 598.

MUELLER, U.W., HAWES, C.S. & JONES, W.R. (1986). Cell surface antigens of human trophoblast: Definition of an apparently unique system with a monoclonal antibody. *Immunol.*, **53**, 135.

O'TOOLE, C.M., POVEY, S. HEPBURN, P. & FRANKS, L.M. (1983). Identity of some human bladder cancer cell lines. *Nature*, **301**, 429.

PATILLO, R.A. & GEY, G.O. (1968). The establishment of human hormone-synthesising cells *in vitro*. *Cancer Res.*, **28**, 1231.

PATILLO, R.A., RUCKERT, A., HUSSA, R., BERNSTEIN, R. & DELFS, E. (1971). The JAr cell line – continuous human multihormone production and controls. *In Vitro*, **6**, 398.

PATEISKY, N., PHILIPP, K., SKODLER, W.D., CZERWENKA, K., HAMILTON, G. & BURCHELL, J. (1985). Radioimmunodetection in patients with suspected ovarian cancer. *J. Nucl. Med.*, **26**, 1369.

PLUMMER, T.H., ELDER, J.H., ALEXANDER, S., PHELAN, A.W. & TARENTINO, A.L. (1984). Demonstration of peptide: N-glycosidase F activity in endo-$\beta$-N-acetylglucosamimidase F preparations. *J. Bio. Chem.*, **259**, 10700.

PULVERTAFT, R.J.V. (1964). Cytology of Burkitt's tumour (African lymphoma). *Lancet*, **i**, 238.

RETTIG, W.J., CORDON-CARDO, C., KOULOS, J.P., LEWIS, J.L., OETTGEN, H.F. & OLD, L.L. (1985). Cell surface antigens of human trophoblast and choriocarcinoma defined by monoclonal antibodies. *Int. J. Cancer*, **35**, 469.

RUOSLAHTI, E. & ENGVALL, E. (1978). Alpha feto-protein. *Scand. J. Immunol.*, **7**, Suppl. 6, 1.

SMITH, N.C., BRUSH, M.G. & LUCKETT, S. (1974). Preparation of human placental villous surface membrane. *Nature*, **252**, 302.

STERN, P.L., GILBERT, P., STERNBERG, S., THOMPSON, S. & CHADA, K. (1984). A monoclonal antibody which detects 125 kDa glycoprotein on embryonal cells and is mitogenic for murine spleen cells. *J. Reprod. Immunol.*, **6**, 313.

STERN, P.L., BERESFORD, N., THOMPSON, S., JOHNSON, P.M. WEBB, P.D. & HOLE, N. (1986). Characterisation of the human trophoblast leukocyte antigenic molecules defined by a monoclonal antibody. *J. Immunol.*, **137**, 1604.

SUNDERLAND, C.A., REDMAN, C.W.G. & STIRRAT, G.M., (1981). Monoclonal antibodies to human syncytiotrophoblast. *Immunol.*, **43**, 541.

SWALLOW, D.M., GENDLER, S., GRIFFITHS, B., CORNEY, G., TAYLOR-PAPADIMITRIOU, J. & BRAMWELL, M.E. (1987). The human tumour-associated epithelial mucins are coded by and expressed hypervariable gene locus PUM. *Nature*, **328**, 82.

TAYLOR-PAPADIMITRIOU, J., PETERSEN, J.A., ARKLIE, J., BURCHELL, J., CERIANI, R.L. & BODMER, W.F. (1981). Monoclonal antibodies to epithelium specific component of the milk fat globule membrane: Production and reaction with cells in culture. *Int. J. Cancer*, **28**, 17.

THOMPSON, S., STERN, P.L., WEBB, M. & 6 others (1984). Differentiation of neuron-like cells and other cell types from cloned human teratocarcinoma cells cultured in retinoic acid. *J. Cell. Sci.*, **72**, 37.

TRAVERS, P. & BODMER, W.F. (1984). Preparation and characterisation of monoclonal antibodies against placental alkaline phosphatase and other human trophoblast associated determinants. *Int. J. Cancer*, **33**, 633.

TROWBRIDGE, I.S., NEWMAN, R.A., DOMINGO, D.L. & SAUVAGE, C. (1984). Transferrin receptor: Structure and function. *Biochem. Pharmacol.*, **33**, 925.

ULLRICH, A., BOLL, J.R., RHEN, E.Y. & 11 others (1985). Human insulin receptor and its relationship to the tyrosine kinase family of oncogenes. *Nature*, **313**, 756.

WALKER, C., DANIELS, M., & MATES, G. (1984). Proliferative response of human venous endothelial cells to medium conditioned by human tumour cells. *Cell. Biol. Int. Rep.*, **8**, 731.

WARR, B.G. & CRUICKSHANK, D.J. (1987). Circulating tumour associated antigen detected by the monoclonal antibody HMFG2 in human epithelial ovarian cancer. *Int. J. Cancer*, **39**, 30.

WATERFIELD, M.D., MAYES, E.L.V. STROOBANT, P. & 5 others (1982). A monoclonal antibody to the human epidermal growth factor receptor. *J. Cell Biochem.*, **20**, 149.

WEBB, P.D., EVANS, P.W., MOLLOY, C.M. & JOHNSON, P.M. (1985). Biochemical studies of human microvillous plasma membrane proteins. *Am. J. Reprod. Immunol. Microbiol.*, **8**, 113.

WILKINSON, M.J.S., HOWELL, A., HARRIS, M., PAD, J.T., SWINDELL, R. & SELLWOOD, R.A. (1984). The prognostic significance of two epithelial membrane antigens expressed by human mammary carcinoma. *Int. J. Cancer*, **33**, 299.

WISEMAN, G., BRAMWELL, M.E., BHAVANANDAN, V.P. & HARRIS, H. (1984). The structure of the Ca-antigen. *Biochem. Soc. Trans.*, **12**, 537.

YAMASHITA, K., NAKAMURA, T., SHIMIZU, T. & OHNO, H. (1986). Monoclonal antibodies to choriocarcinoma. *Am. J. Reprod. Immunol. Microbiol.*, **11**, 130.

ZEUTHEN, J. NOGAARD, J.O.R., AVNER, P. & 5 others (1980). Characterisation of an ovarian teratocarcinoma cell line. *Int. J. Cancer*, **25**, 19.